# Iatrogenic Brachial Artery Injury During Long Proximal Humeral Interlocking System (PHILOS) Plating

**DOI:** 10.7759/cureus.68413

**Published:** 2024-09-01

**Authors:** Rahul Salunkhe, Bhushan R Patil, Dattatray Bhakare, Amit C Patil, Faiz R Pervez, Ishan Shevate

**Affiliations:** 1 Orthopaedics, Dr. D. Y. Patil Medical College, Hospital and Research Center, Dr. D. Y. Patil Vidyapeeth (Deemed to be University), Pune, IND; 2 Plastic Surgery, Dr. D. Y. Patil Medical College, Hospital and Research Center, Dr. D. Y. Patil Vidyapeeth (Deemed to be University), Pune, IND

**Keywords:** postoperative complications, iatrogenic disease, bone plates, humeral fractures, brachial artery injuries

## Abstract

Iatrogenic vascular injuries are rare but potentially serious complications that can occur during orthopedic procedures involving the proximal humerus. We present a case report of a patient who sustained a brachial artery injury during a long Proximal Humeral Interlocking System (PHILOS) plating procedure for a proximal humeral fracture. A 62-year-old female patient with a left humerus proximal 1/3 shaft fracture underwent open reduction and internal fixation with a long PHILOS plate. During the procedure, difficulty was encountered in achieving adequate plate positioning due to osteoporotic bone and fracture comminution. Upon insertion of a distal second last screw, brisk brachial artery bleeding was encountered. Immediate hemostasis measures were taken, and a plastic surgeon was consulted. The brachial artery injury was identified and repaired with a cephalic vein graft harvested and flushed. Postoperatively, the patient developed median nerve neuropraxia. This case highlights the risk of iatrogenic brachial artery injury during left humerus proximal 1/3 shaft fracture fixation, especially in cases with technical challenges due to osteoporotic bone or comminution. Prompt recognition, involvement of vascular surgery, and appropriate management are crucial in mitigating potential devastating consequences. Associated neurological complications, such as nerve injuries, can also occur and should be monitored. Meticulous surgical technique, anatomical awareness, and vigilant monitoring are essential to minimize the risk of vascular and neurological complications during these procedures. Iatrogenic brachial artery injury is a rare but potentially serious complication of humerus proximal 1/3 shaft fracture. Early recognition, multidisciplinary involvement, and appropriate management strategies are crucial in optimizing patient outcomes and preventing long-term morbidity.

## Introduction

Iatrogenic vascular injuries during orthopedic procedures, although uncommon, can lead to significant morbidity and potential long-term consequences [1]. These injuries can occur due to anatomical variations, inadvertent trauma, or technical challenges during the surgical intervention [2]. One such complication is brachial artery injury, which can arise during procedures involving the proximal humerus, such as fixation with the Proximal Humeral Interlocking System (PHILOS) plate [3]. The PHILOS plate is a widely used implant for the treatment of complex proximal humeral fractures, providing stable fixation and facilitating early mobilization [4]. However, the surgical approach and placement of this long plate can be technically demanding, particularly in cases with extensive comminution or osteoporotic bone. The close proximity of the brachial artery to the surgical site increases the risk of iatrogenic injury, which can have potentially devastating consequences if not recognized and managed promptly [5]. Brachial artery injuries can manifest as active bleeding, thrombosis, or pseudoaneurysm formation, leading to ischemic complications and potential limb-threatening situations [6]. Early recognition of these injuries is crucial, as delayed diagnosis and treatment can result in increased morbidity, prolonged hospital stays, and potential long-term disability. This case report aims to highlight the occurrence of an iatrogenic brachial artery injury during a long PHILOS plating procedure for a proximal humeral 1/3 shaft fracture. It underscores the importance of meticulous surgical technique, anatomical awareness, and vigilant monitoring for potential vascular complications during orthopedic procedures involving the proximal humerus.

## Case presentation

Patient information

A 62-year-old female patient presented with pain and swelling over the left upper arm for the past 10 days. She had sustained a low-energy fall at home 10 days prior. She was diagnosed with a left humeral proximal 1/3 shaft fracture.

Clinical findings

**Figure 1:**
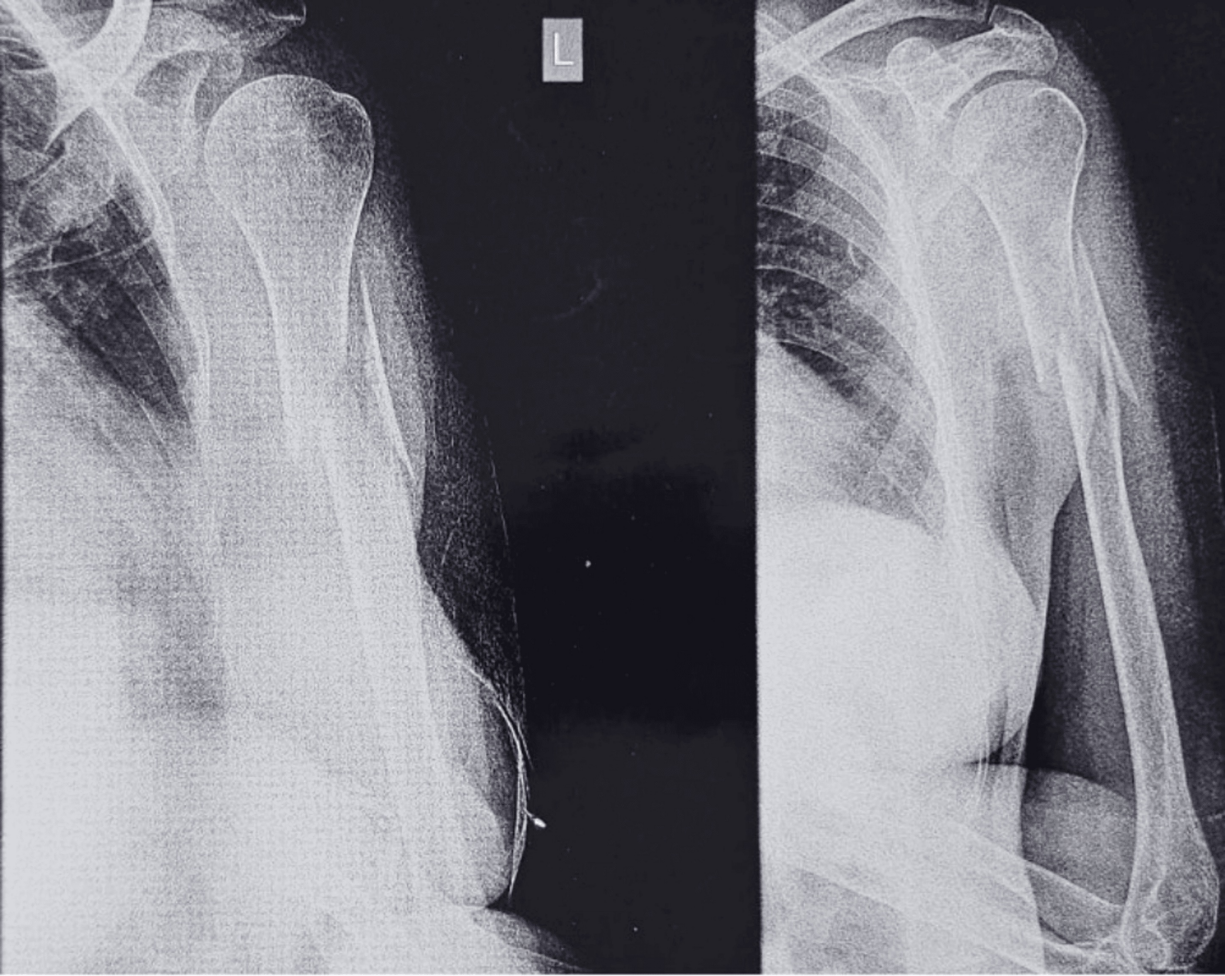
Pre-operative X-ray showing left humerus proximal 1/3 shaft fracture.

Physical examination revealed swelling, tenderness, and deformity of the left proximal humerus, with intact distal neurovascular status. The radiographic evaluation confirmed a left humeral proximal 1/3 shaft fracture. A bone mineral density (BMD) scan revealed that the patient had osteoporosis (Figure 1).

Therapeutic intervention

**Figure 2:**
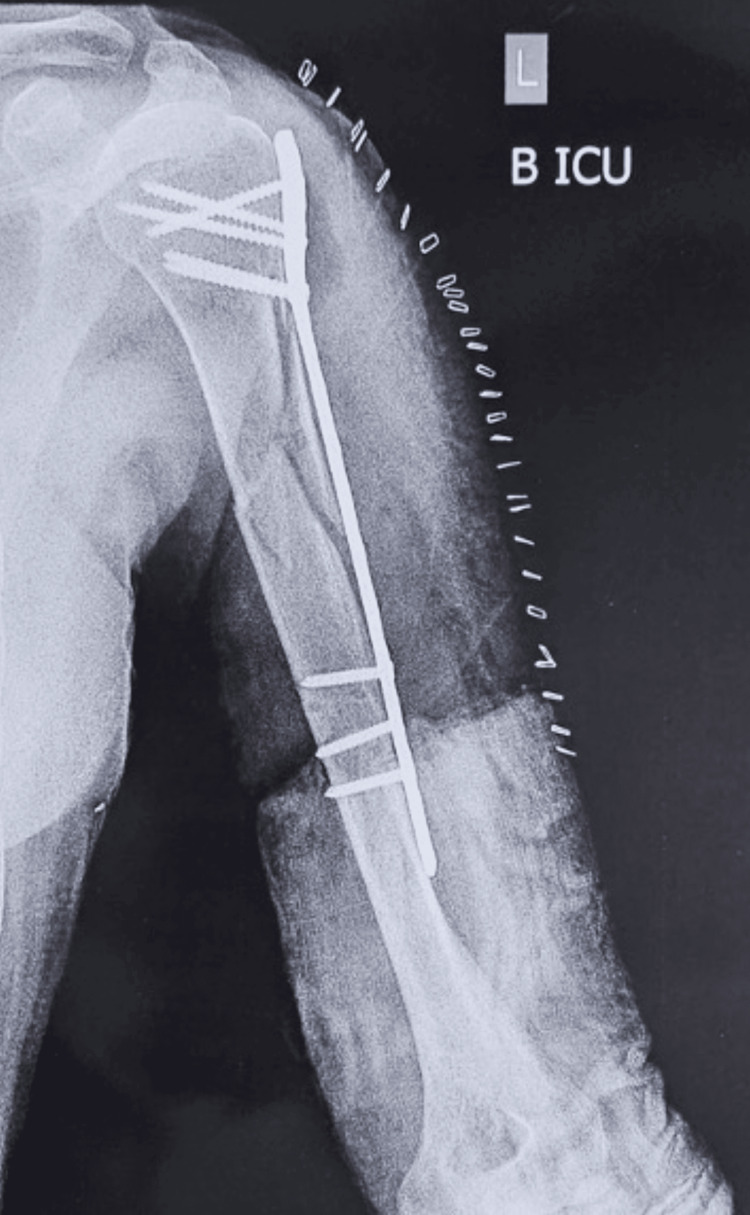
Postoperative X-ray.

The patient was scheduled for left open reduction and internal fixation with a long PHILOS plate. The approach used was deltoid splitting extended to the shaft. The procedure was performed under general anesthesia utilizing a deltoid-splitting approach (Figure 2).

**Figure 3:**
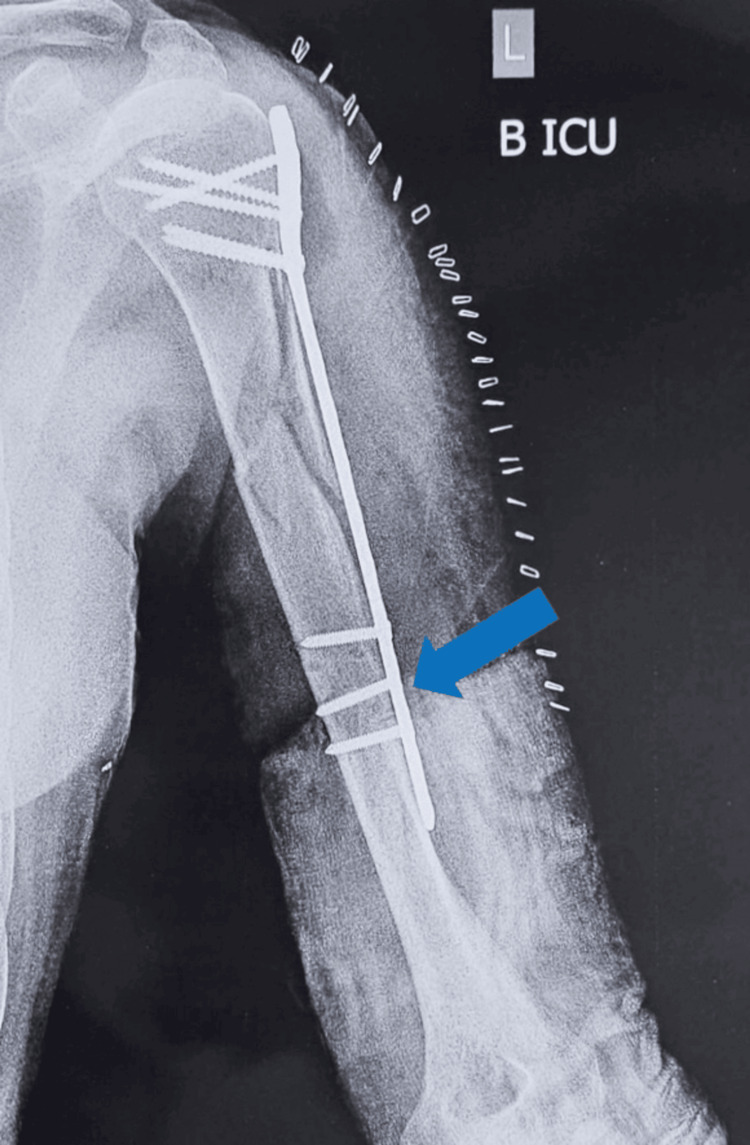
X-ray of left humerus anterior-posterior view. The blue arrow in the figure indicates the site of brachial artery iatrogenic injury.

Fracture reduction and plate placement were achieved according to standard surgical techniques. During the procedure, difficulty was encountered in obtaining adequate plate positioning due to the osteoporotic nature of the bone and comminution at the fracture site. After securing the plate to the humeral shaft, proximal locking screws were placed. Upon drilling the second-to-last screw from the distal end, a gush of blood was observed from the surgical field, and brisk brachial artery bleeding was encountered (Figure 3).

Immediate measures were taken to achieve hemostasis through direct pressure. A plastic surgeon was consulted, and the brachial artery injury was identified and repaired primarily with a cephalic vein graft.

A nine-hole plate was used, which was fixed proximally with five locking screws and distally with one cortical screw. The injury occurred while drilling the second-to-last hole from the distal end on the nine-hole PHILOS plate.

Brachial artery repair

**Figure 4:**
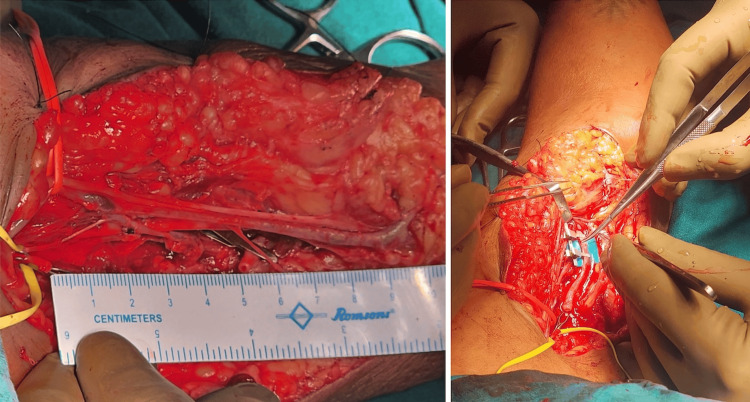
Cephalic vein graft was harvested.

**Figure 5:**
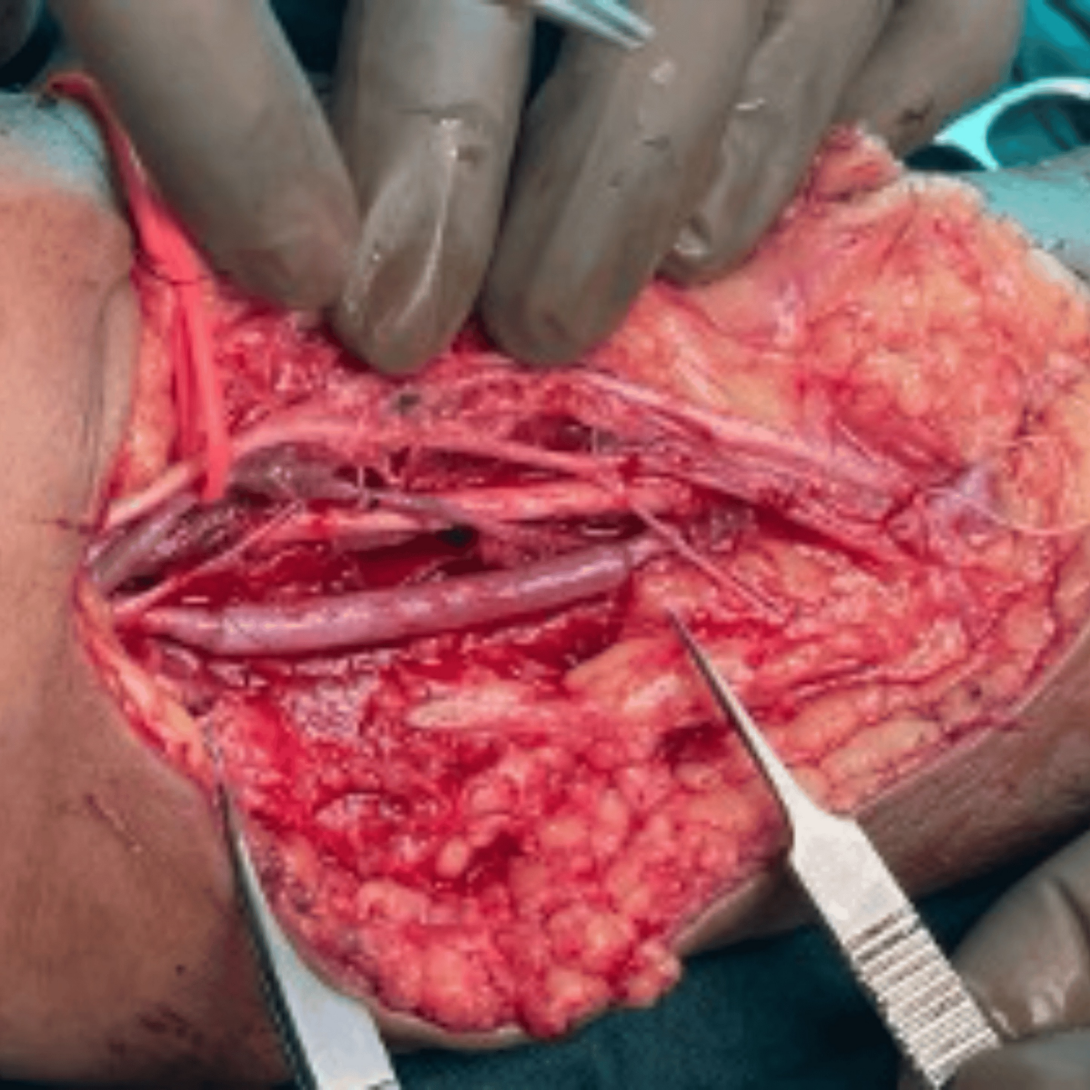
Brachial artery anastomosis with cephalic vein graft.

The limb was found to be pulseless; the radial and brachial pulses were not palpable. The wound was packed with sterile gauze, the limb was elevated, and an Esmarch bandage was applied. Plastic surgery was informed and involved. A medial incision was made over the suspected brachial artery injury site. Superficial and deep dissection was performed, and neurovascular structures were identified and isolated. The median nerve was identified. The brachial artery was traced to the site of trauma, approximately 10 cm proximal to the elbow joint. The extent of the intimal injury was identified with a thrombus. A large graft was used, and the injured vessel was excised. The defect measured 5 cm. A cephalic vein graft was harvested, flushed, and used for the brachial artery anastomosis, performed distally and then proximally with 8-0 Ethilon sutures. Clamps were removed, and adequate pulsation was observed; the radial pulse was palpable, and the fingers were well-perfused (Figure 4 and Figure 5).

Postoperative course

Following the vascular repair, the patient's distal vascular status remained intact. The postoperative radial pulse was palpable, and finger movements were present. Neuropraxia of the median nerve with an ape hand deformity was noted. The patient was started on aspirin 75 mg for three weeks. The patient was monitored closely in the intensive care unit for 48 hours and subsequently transferred to the orthopedic ward. Anticoagulation therapy was initiated to prevent graft thrombosis. The patient was followed up at regular intervals, and at the six-week follow-up, the patient had a complete recovery of neuropraxia. Serial Doppler ultrasound examinations confirmed the patency of the brachial artery graft.

## Discussion

Iatrogenic vascular injuries are rare but potentially devastating complications that can occur during orthopedic procedures, particularly those involving the proximal humeral shaft and the surrounding vascular structures. This case report highlights the occurrence of a brachial artery injury during a long PHILOS plating procedure for a proximal humeral shaft fracture. The incidence of iatrogenic brachial artery injury during proximal humeral shaft fracture fixation has been reported to range from 0.5% to 2% [7]. While the PHILOS plate is widely used for the treatment of complex proximal humeral fractures, its long and anatomically contoured design can pose technical challenges, especially in cases with osteoporotic bone or extensive comminution [4]. Several factors may have contributed to the brachial artery injury in our case, including the osteoporotic nature of the bone, fracture comminution, and the need for technical maneuvers during plate positioning and screw insertion. These challenges are not uncommon, as reported by Wijgerde et al. in their systematic review, which found a high rate of complications associated with PHILOS plating, including vascular injuries [7].

The prompt recognition and management of the brachial artery injury in our case were crucial in preventing potential limb-threatening complications. Early involvement of plastic surgery and the decision to repair the artery with a cephalic vein graft were in line with the recommended management strategies for such injuries [2,6]. Interestingly, our case also demonstrates the potential for associated neurological complications, as evidenced by the median nerve neuropraxia observed in the postoperative period. While vascular injuries are more commonly reported, nerve injuries can also occur during proximal humeral fracture fixation, with reported incidences ranging from 6.2% to 67% [8,9].

In this case, we performed the surgery in a single sitting as the vascular injury needed to be addressed immediately. To prevent such injuries, adjustments should be made to the length of the drill according to the sleeve length and width of the humerus. While the occurrence of iatrogenic vascular injuries during orthopedic procedures is relatively rare, our case emphasizes the importance of meticulous surgical technique, anatomical awareness, and vigilant monitoring for potential complications. Early recognition and prompt management, often involving a multidisciplinary approach, are crucial in mitigating the potentially devastating consequences of such injuries.

## Conclusions

This case report underscores the potential for iatrogenic vascular injuries during orthopedic procedures, particularly those involving complex proximal humeral fractures and the use of PHILOS plating systems. Although such injuries are rare, they can have serious, limb-threatening consequences if not promptly recognized and managed. Our experience highlights the critical importance of meticulous surgical technique, anatomical awareness, and vigilant monitoring to mitigate these risks. The early involvement of a multidisciplinary team, including vascular surgery, is essential for the effective management and repair of such injuries. Additionally, adjusting surgical techniques and tools, such as drill length, based on anatomical considerations and fracture complexity can help prevent similar complications. This case serves as a reminder of the need for heightened awareness and preparedness to address potential vascular and neurological complications, ensuring optimal outcomes and minimizing long-term consequences for patients undergoing complex orthopedic procedures.
